# Association of inflammation/nutrition-based indicators with Parkinson’s disease and mortality

**DOI:** 10.3389/fnut.2024.1439803

**Published:** 2024-09-17

**Authors:** Huafang Jia, Kaixiang Yin, Jihu Zhao, Fengyuan Che

**Affiliations:** ^1^Department of Medicine, Qingdao University, Qingdao, Shandong, China; ^2^Department of Emergency Medicine, The Affiliated Hospital of Qingdao University, Qingdao, China; ^3^Department of Neurosurgery, The Affiliated Hospital of Qingdao University, Qingdao, Shandong, China; ^4^Shandong Provincial Clinical Research Center for Geriatric Diseases, Key Laboratory of Neurophysiology, Health Commission of Shandong Province, Linyi Key Laboratory of Neurophysiology, Key Laboratory for Translational Oncology, Department of Neurology, Linyi People’s Hospital, Xuzhou Medical University, Linyi, Shandong, China

**Keywords:** inflammation, nutrition, Parkinson’s disease, mortality, NHANES

## Abstract

**Objective:**

The study explores the association between inflammation/nutrition-based indicators, Parkinson’s disease (PD), and all-cause mortality among adult participants.

**Methods:**

The analysis included 38,091 participants from National Health and Nutrition Examination Survey (NHANES) 1999–2018. Inflammation/nutrition-based indicators were derived from a comprehensive set of parameters, including neutrophil-albumin ratio (NAR), prognostic nutritional index (PNI), monocyte-albumin ratio (MAR), red cell distribution width-albumin ratio (RAR), hemoglobin, albumin, lymphocyte, and platelet (HALP) score, advanced lung cancer inflammation index (ALI), geriatric nutrition risk index (GNRI), and controlling nutritional status (CONUT) score. PD status was determined based on self-reported anti-parkinsonian medication use. Mortality data were obtained from the National Death Index, linked up to December 30, 2019.

**Results:**

After multivariate adjustment, all inflammation/nutrition-based indicators showed significant associations with all-cause mortality among adult participants. The random survival forest emphasized the importance of inflammation/nutrition-based indicators and their components in predicting mortality, with PNI and RAR being the most important indicators for predicting all-cause mortality. Individuals with PD have a significantly higher risk of all-cause mortality compared to those without PD (HR = 1.747 [1.363–2.238], *P* < 0.001) among adults. Additionally, elevated levels of inflammation/nutrition-based indicators are associated with a higher risk of all-cause mortality among individuals with PD compared to those without PD. This suggested a synergistic effect of PD and elevated levels of inflammatory/nutritional indicators on mortality risk. Specifically, individuals with PD and elevated NAR (HR = 2.066 [1.398–3.052], *P* < 0.001), MAR (HR = 2.249 [1.612–3.138], *P* < 0.001), RAR (HR = 1.617 [1.179–2.218], *P* = 0.003), ALI (HR = 1.763 [1.225–2.537], *P* = 0.002) and CONUT (HR = 2.221 [1.434–3.440], *P* < 0.001), and not elevated PNI (HR = 1.771 [1.295–2.423], *P* < 0.001), HALP (HR = 1.738 [1.242–2.432], *P* = 0.001), and GNRI (HR = 2.689 [1.898–3.811], *P* < 0.001) have a substantially higher risk of mortality compared to those without PD.

**Conclusion:**

Inflammation and nutrition status play crucial roles in predicting all-cause mortality among adults, particularly in the context of PD. The study underscores the importance of considering both factors in mortality risk assessment and provides valuable insights for future research and clinical practice.

## Introduction

Parkinson’s disease (PD) is the second most prevalent neurodegenerative disorder, which is characterized by the progressive loss of dopaminergic neurons in the brain, leading to a range of motor and non-motor symptoms (1). The incidence of Parkinson’s disease is estimated to be around 14 per 100,000 in the general population (2). As populations worldwide experience demographic shifts towards older age groups, the number of individuals at risk for PD naturally increases (3). The primary clinical manifestations of PD encompass a range of motor symptoms, such as tremor, rigidity, akinesia/bradykinesia, postural Instability, and non-motor symptoms, including cognitive impairment, mood disorders, sleep disturbances, autonomic dysfunction, sensory symptoms (4). The combination of motor and non-motor symptoms in PD can significantly impact the quality of life of affected individuals (4). The individuals with PD had a higher all-cause mortality compared to the general population, particularly in individuals with dementia co-morbidity (5).

Inflammatory and nutritional status contribute to the development and progression of various chronic diseases and conditions, including PD. The role of the inflammatory response in PD pathogenesis and development is significant. Currently, many of studies highlighted the role of neuroinflammation in the progression of the disease (6). Neuroinflammation, characterized by the activation of microglia and the release of inflammatory biomarkers in the brain, appears to play a significant role in the progression of PD pathology. Researchers are exploring the integration of multiple biomarkers, including both inflammatory and non-inflammatory markers, to enhance predictive value (7). As a marker of inflammation in clinical practice and research studies, elevated C-reactive protein (CRP) levels have been observed in PD patients (8). Numerous clinical researches have also highlighted the importance of good nutritional status in PD patients for improving various aspects of the condition (9). The nutritional status of PD patients has been closely linked to both motor and non-motor symptoms. Malnutrition could be a significant risk factor in PD patients, for example, a lower body mass index (BMI) has been associated with a worse survival outcome (10). A recent study found some molecular biomarkers were related with the clinical severity of PD, such as body weight, BMI, and levels of hemoglobin, cholesterol, and high-density lipoprotein (HDL), which could monitor the nutritional condition and the progression of PD (9). Moreover, lower hemoglobin levels and albumin levels have been reported to be related to the severity of PD (11, 12).

Combining multiple biomarkers can enhance prognostic accuracy compared to using individual biomarkers alone. Several studies have explored the potential of combining various biomarkers to predict the progression of PD (13). It’s advisable to utilize novel biomarkers with clinical blood samples in the prognosis of patients with PD. For example, research suggested that the C-reactive protein-albumin ratio may be a more comprehensive marker of both inflammation and nutritional status compared to using CRP or albumin alone (14).

While some of the inflammation and nutrition-based indicators discussed in our study have been previously mentioned in the context of various diseases, many of these biomarkers are not commonly used to assess mortality risk specifically in PD. Moreover, we need to select valuable biomarkers from large scale screens which could be correlated with the survival outcomes in adults and patients with PD. There is a lack of study specifically for individuals with PD on the relationships between inflammatory and nutritional biomarkers and the mortality risk of PD. To select some effective indicators, we aimed to explore the association between inflammation/nutrition-based indicators and PD and all-cause mortality among adult participants. Specific inflammation/nutrition-based indicators included neutrophil-albumin ratio (NAR), prognostic nutritional index (PNI), monocyte-albumin ratio (MAR), red cell distribution width-albumin ratio (RAR), hemoglobin, albumin, lymphocyte, and platelet (HALP) score, advanced lung cancer inflammation index (ALI), geriatric nutrition risk index (GNRI), and controlling nutritional status (CONUT) score.

## Materials and methods

### Study population

The NHANES is a program conducted by the National Center for Health Statistics (NCHS), which is part of the Centers for Disease Control and Prevention (CDC) in the United States (15). This survey is designed to assess the health and nutritional status of adults and children in the United States through interviews, physical examinations, and laboratory tests. It employs a complex, multistage probability sampling strategy to select participants from various demographic and geographic strata across the United States. This sampling approach ensures that the survey results can be generalized to the entire U.S. population. The research protocols received approval from the NCHS Research Ethics Review Board, with all participants providing written informed consent.

A total of 101,316 participants from the NHANES spanning the years 1999 to 2018 were initially identified. Exclusions were made for individuals with missing information on PD assessment (*n* = 109), those aged under 20 (*n* = 46,210), and those with missing data for inflammation and nutritional indicators (n = 7,120). Pregnant participants (*n* = 1,289) and those with missing covariate data (*n* = 8,462) were also excluded. After these exclusions, 38,126 participants were enrolled. During follow-up and ineligibility assessment, an additional 35 individuals were deemed ineligible. The final eligible study population comprised 38,091 participants for subsequent analysis (Supplementary Figure 1).

### Assessment of inflammation and nutritional indicators

In this study, we evaluated inflammation and nutritional indicators using a comprehensive set of parameters including neutrophil, lymphocyte, monocyte, hemoglobin, RDW, serum albumin, total cholesterol, and BMI. CBC parameters were derived using the Beckman Coulter methodology, which involves an automatic diluting and mixing device for sample processing and a single-beam photometer for hemoglobinometry. The Beckman Coulter DxH 800 instrument at the NHANES mobile examination center (MEC) analyzed all blood specimens. Total cholesterol levels were enzymatically measured, where esterified cholesterol undergoes conversion to cholest-4-en-3-one and hydrogen peroxide. The subsequent reaction with 4-aminophenazone in the presence of peroxidase produces a colored product specifically measured at 505 nm. Albumin concentration was measured using bromcresol purple (BCP) dye, inducing a color change when binding with albumin within a specific pH range, measured at 600 nm. BMI was calculated as weight in kilograms divided by height in meters squared, rounded to one decimal place. For further details on laboratory methods employed, refer to the Laboratory Method Files section at https://wwwn.cdc.gov/nchs/nhanes/continuousnhanes/labmethods.aspx?Cycle=2017–2018.

Based on the previously mentioned Inflammation and Nutritional Indicators, we derived a set of composite indicators reflecting both inflammation and nutrition status. These inflammation/nutrition-based indicators include NAR, PNI, MAR, RAR, HALP Score, ALI, GNRI, and CONUT Score (16–23). These composite indicators are derived from various combinations of the individual inflammation and nutritional parameters mentioned earlier. The calculation methods for the combination of each nutrition/inflammation-based indicator are shown in Supplementary Table 1.

### Assessment of PD

The determination of PD status in this study was based on participants’ self-reported use of medication specifically indicated for treating PD (24). Participants were categorized as having PD if they reported actively receiving treatment with anti-parkinsonian medication. Specifically, PD was defined if individuals reported using medications such as Benztropine, Methyldopa, Carbidopa, Levodopa, Entacapone, Amantadine, and Ropinirole (25). Conversely, participants who did not report taking any anti-parkinsonian medication were classified as not having PD.

### Assessment of mortality

The assessment of mortality involves linking participant data with the National Death Index (NDI) in the NHANES 1999–2018. The NDI is a centralized database in the United States that compiles mortality data from various sources, including death certificates, to provide comprehensive information on deaths nationwide. This linkage ensures accurate determination of participant deaths, with meticulous maintenance of mortality records up to December 31, 2019.

### Other covariates

The covariates considered in this study include demographic factors, lifestyle behaviors, and health indicators. Demographic information comprises age (grouped as 20–39, 40–59, or ≥ 60 years), sex (male or female), race/ethnicity (categorized as non-Hispanic White, non-Hispanic Black, or other race), living status (married/living with partner or single/divorced/widowed), and education level (below high school, high school, or above high school). Additionally, family poverty-income ratio (PIR) was evaluated, categorized as ≤ 1.0, 1.1–3.0, or > 3.0. Lifestyle behaviors encompass drinking status (nondrinker, low-to-moderate drinker, or heavy drinker), smoking status (never smoker, former smoker, or current smoker), and physical activity levels (inactive, insufficiently active, or active) (26). Dietary quality was assessed using the Healthy Eating Index (HEI), divided into quartiles, while comorbidity burden was evaluated using the Charlson Comorbidity Index (CCI), treated as a continuous variable. Detailed definitions for family PIR, smoking status, drinking status, physical activity levels, HEI, and CCI are provided in the methodology section of the Supplementary materials.

### Statistical analysis

To ensure the robustness of national estimations, adherence to guidelines stipulated by the National Center for Health Statistics was maintained, incorporating primary sampling units, sample weights, and strata during the process of data analysis. Weighted analyses were executed utilizing the “survey” package within the R statistical software. Continuous variables were described as mean (standard error) for those with a normal distribution and as median [interquartile range] for those with a non-normal distribution. For comparisons between groups, we used Student’s t-test for continuous variables with a normal distribution. For continuous variables that did not exhibit a normal distribution, we employed the Mann-Whitney U test. Categorical variables were represented as numerical values (percentages). Unordered categorical variables underwent chi-square testing, whereas ordered categorical variables were assessed utilizing the Kruskal-Wallis H test. Missing data were imputed employing the “mice” package in conjunction with the random forest algorithm.

Inflammation/nutrition-based indicators were classified into two groups based on the median, with participants below the median used as the reference in the regression model. Logistic regression analysis was conducted to investigate the relationship between inflammation/nutrition-based indicators and the prevalence of PD, yielding odds ratios (ORs) and corresponding 95% confidence intervals (CIs). Survival analysis was performed using the log-rank test coupled with Kaplan-Meier survival curves. COX proportional hazard regression was employed to evaluate the association of PD with all-cause mortality, as well as the impact of inflammation/nutrition-based indicators on mortality outcomes among participants diagnosed with PD. Hazard ratios (HRs) and 95% CIs were reported, with the assessment of assumptions conducted through Schoenfeld residuals and examination of multicollinearity using variance inflation factors (VIF). Furthermore, a restricted cubic spline curve was utilized to explore potential non-linear relationships between inflammation/nutrition-based indicators and all-cause mortality in the adult population.

Time-dependent receiver operating characteristic (ROC) curves were utilized to assess the predictive capacity of inflammation/nutrition-based indicators for mortality, and the area under the curve (AUC) was evaluated accordingly. Spearman’s correlation analysis was conducted to compute correlation coefficients between inflammation/nutrition-based indicators and their constituent components. Inflammatory indicators demonstrating significant predictive characteristics for mortality were identified through the random survival forest method, which furnishes measures of feature importance. Statistical analyses were carried out using R software (version 4.3.2), with statistical significance defined as *P* < 0.05.

## Results

### Baseline characteristics of the participants

Table 1 displays baseline characteristics of adult NHANES 1999–2018 participants, categorized by all-cause mortality. Of the 38,091 participants analyzed, 5,776 experience all-cause mortality. Significant differences were observed in age distribution (*P* < 0.001), with higher proportions of individuals aged 40–59 years and ≥ 60 years in the mortality group. Sex distribution also differed significantly (*P* < 0.001), with more males in the mortality group. Statistically significant differences (all *P* < 0.001) were noted in race/ethnicity, marital status, education level, family poverty-income ratio, smoking status, drinking status, and physical activity levels between participants with and without all-cause mortality. While all other inflammation and nutritional indicators showed statistically significant differences between the two groups, BMI did not exhibit any significant difference. Similarly, inflammation/nutrition-based indicators (including NAR, PNI, MAR, RAR, HALP score, ALI, GNRI, and CONUT score) showed significant differences between the two groups.

**TABLE 1:** Baseline characteristics of adult participants according to all-cause mortality in NHANES 1999–2018.

Characteristics	Total	All-cause mortality		*P*-value
		No (*n* = 32315)	Yes (*n* = 5776)	
Age, %				< 0.001
20–39 years	12420 (36.62)	12207 (40.23)	213 (6.16)	
40–59 years	12519 (38.43)	11628 (40.31)	891 (22.52)	
≥ 60 years	13152 (24.95)	8480 (19.46)	4672 (71.32)	
Sex, %				< 0.001
Female	18869 (50.52)	16376 (50.85)	2493 (47.73)	
Male	19222 (49.48)	15939 (49.15)	3283 (52.27)	
Race/ethnicity, %				< 0.001
Non-Hispanic White	18033 (71.13)	14419 (70.04)	3614 (80.31)	
Non-Hispanic Black	7505 (9.93)	6452 (9.95)	1053 (9.77)	
Other race	12553 (18.94)	11444 (20.01)	1109 (9.92)	
Marital status, %				< 0.001
Married/living with partner	14974 (35.56)	12289 (34.60)	2685 (43.68)	
Single/divorced/widowed	23117 (64.44)	20026 (65.40)	3091 (56.32)	
Education level, %				< 0.001
Below high school	9572 (15.84)	7367 (14.21)	2205 (29.62)	
High school	8911 (24.18)	7431 (23.69)	1480 (28.33)	
Above high school	19608 (59.98)	17517 (62.10)	2091 (42.05)	
Family PIR, %				< 0.001
≤ 1.0	7470 (13.38)	6322 (13.14)	1148 (15.42)	
1.1–3.0	16048 (35.76)	13032 (34.19)	3016 (49.01)	
> 3.0	14573 (50.86)	12961 (52.67)	1612 (35.57)	
Smoking status, %				< 0.001
Never smoker	20236 (53.26)	17967 (55.08)	2269 (38.08)	
Former smoker	9793 (25.31)	7475 (23.80)	2318 (38.11)	
Current smoker	8043 (21.40)	6857 (21.12)	1186 (23.80)	
Drinking status, %				< 0.001
Nondrinker	8546 (18.24)	6865 (17.18)	1681 (27.24)	
Low-to-moderate drinker	26263 (71.68)	22702 (72.85)	3561 (61.78)	
Heavy drinker	3282 (10.08)	2748 (9.97)	534 (10.98)	
Physical activity, %				< 0.001
Inactive	9934 (20.99)	7459 (18.79)	2475 (39.52)	
Insufficiently active	14405 (40.78)	12486 (41.44)	1919 (35.17)	
Active	13752 (38.23)	12370 (39.76)	1382 (25.31)	
HEI-2015 score	49.53 (40.47, 59.37)	49.36 (40.28, 59.27)	50.96 (41.98, 60.29)	< 0.001
CCI	0.86 (0.01)	0.74 (0.01)	1.87 (0.03)	< 0.001
Parkinson’s disease, %				< 0.001
No	37741 (99.18)	32080 (99.31)	5661 (98.08)	
Yes	350 (0.82)	235 (0.69)	115 (1.92)	
**Inflammation and nutritional indicators**				
Neutrophil, 10^3^/μL	4.00 (3.20, 5.10)	4.00 (3.10, 5.10)	4.30 (3.30, 5.40)	< 0.001
Lymphocyte, 10^3^/μL	2.00 (1.60, 2.50)	2.00 (1.70, 2.50)	1.90 (1.40, 2.40)	< 0.001
Monocyte, 10^3^/μL	0.50 (0.40, 0.70)	0.50 (0.40, 0.70)	0.60 (0.50, 0.70)	< 0.001
Hemoglobin, g/dL	14.40 (13.40, 15.40)	14.40 (13.50, 15.40)	14.20 (13.10, 15.20)	< 0.001
RDW, %	12.80 (12.30, 13.50)	12.70 (12.30, 13.40)	13.00 (12.40, 13.80)	< 0.001
Serum albumin, g/L	43.00 (41.00, 45.00)	43.00 (41.00, 45.00)	42.00 (40.00, 44.00)	< 0.001
Total cholesterol, mg/dL	193.00 (167.00, 221.00)	193.00 (167.00, 221.00)	196.00 (167.00, 225.00)	0.004
Body mass index, kg/m^2^	27.71 (24.10, 32.22)	27.71 (24.10, 32.25)	27.74 (24.20, 32.12)	0.970
**Inflammation/nutrition-based indicators**				
NAR	0.09 (0.07, 0.12)	0.09 (0.07, 0.12)	0.10 (0.08, 0.13)	< 0.001
PNI	53.50 (50.50, 56.50)	53.50 (50.50, 56.50)	51.50 (48.00, 55.00)	< 0.001
MAR	0.01 (0.01, 0.02)	0.01 (0.01, 0.02)	0.01 (0.01, 0.02)	< 0.001
RAR	0.30 (0.28, 0.32)	0.30 (0.28, 0.32)	0.31 (0.29, 0.34)	< 0.001
HALP score	50.57 (39.00, 65.49)	51.03 (39.63, 65.82)	45.82 (33.32, 62.45)	< 0.001
ALI	239.44 (174.59, 327.86)	236.16 (173.25, 321.42)	270.07 (191.12, 388.47)	< 0.001
GNRI	120.18 (113.30, 128.97)	120.34 (113.47, 129.13)	118.66 (111.21, 127.60)	< 0.001
CONUT score	0.00 (0.00, 1.00)	0.00 (0.00, 1.00)	1.00 (0.00, 1.00)	< 0.001

HEI-2015, Healthy Eating Index 2015; CCI, Charlson Comorbidity Index; RDW, red cell distribution width; NAR, neutrophil-albumin ratio; PNI, prognostic nutritional index; MAR, monocyte-albumin ratio; RAR, red cell distribution width-albumin ratio; HALP, hemoglobin, albumin, lymphocyte, and platelet; ALI, advanced lung cancer inflammation index; GNRI, geriatric nutrition risk index; CONUT, controlling nutritional status. Normally distributed continuous variables are described as means ± SEs, and continuous variables without a normal distribution are presented as medians [interquartile ranges]. Categorical variables are presented as numbers (percentages). N reflect the study sample while percentages reflect the survey-weighted data.

Supplementary Table 2 presents baseline characteristics of adult participants stratified by the presence or absence of PD. Of the 38,091 participants included in the analysis, 37,741 were categorized as not having PD, while 350 were identified as having the condition. Significant disparities were noted in various inflammation and nutritional indicators between the two groups. Specifically, differences were observed in lymphocyte count, hemoglobin, RDW, serum albumin levels, and inflammation/nutrition-based indicators (NAR, PNI, HALP score, ALI) among individuals with and without PD. These variations indicate potential links between PD and changes in inflammation and nutritional status.

### The relationship between inflammation/nutrition-based indicators and PD

Table 2 presents the logistic regression analysis examining the association between inflammation/nutrition-based indicators and the prevalence of PD among adult. Adjusting for various demographic and lifestyle factors in Model 2, statistically significant associations persisted for the PNI (OR = 0.702 [0.515, 0.957], P = 0.026) and RAR (OR = 1.872 [1.320, 2.655], *P* < 0.001) when comparing participants above the median with those below the median. Conversely, no significant associations were observed for the NAR, MAR, HALP score, ALI, GNRI, and CONUT score.

**TABLE 2:** Logistic regression analysis of the relationship between inflammation/nutrition-based indicators and the prevalence of Parkinson’s disease among adults in NHANES 1999–2018.

	Crude		Model 1		Model 2	
	OR (95% CI)	*P*-value	OR (95% CI)	*P*-value	OR (95% CI)	*P*-value
NAR	1.389(1.055, 1.828)	0.019	1.345 (1.016, 1.780)	0.039	1.126 (0.837, 1.515)	0.429
PNI	0.534 (0.401, 0.712)	< 0.001	0.689 (0.513, 0.925)	0.014	0.702 (0.515, 0.957)	0.026
MAR	1.036 (0.787, 1.364)	0.800	0.972 (0.737, 1.281)	0.838	0.853 (0.640, 1.137)	0.276
RAR	2.667 (1.918, 3.709)	< 0.001	2.245 (1.594, 3.162)	< 0.001	1.872 (1.320, 2.655)	< 0.001
HALP score	0.723 (0.545, 0.959)	0.025	0.860 (0.651, 1.138)	0.289	0.826 (0.617, 1.106)	0.198
ALI	1.577 (1.173, 2.122)	0.003	1.438 (1.071, 1.932)	0.016	1.300 (0.962, 1.758)	0.088
GNRI	1.062 (0.807, 1.397)	0.665	1.144 (0.858, 1.525)	0.358	1.088 (0.814, 1.455)	0.566
CONUT score	1.233 (0.949, 1.601)	0.117	1.155 (0.883, 1.510)	0.291	1.050 (0.806, 1.369)	0.715

NAR, neutrophil-albumin ratio; PNI, prognostic nutritional index; MAR, monocyte-albumin ratio; RAR, red cell distribution width-albumin ratio; HALP, hemoglobin, albumin, lymphocyte, and platelet; ALI, advanced lung cancer inflammation index; GNRI, geriatric nutrition risk index; CONUT, controlling nutritional status; Inflammation/nutrition-based indicators were classified into two groups based on the median, with participants below the median used as the reference in the regression model. Model 1: Adjusted for age (20–39, 40–59, or ≥ 60 years), sex (male or female), and race/ethnicity (non-Hispanic White, non-Hispanic Black or other race); Model 2: Model 1 + marital status (married/living with partner, or single/divorced/widowed), education level (below high school, high school, or above high school), family PIR ( ≤ 1.0, 1.1–3.0, or > 3.0), drinking status (nondrinker, former drinker, or current drinker), smoking status (never smoker, former smoker, or current smoker, physical activity (inactive, insufficiently active, or active), HEI (in quartiles), and CCI (continous).

Supplementary Table 3 presents the logistic regression analysis investigating the relationship between inflammation and nutritional indicators and the prevalence of PD among adult. Adjusting for various demographic and lifestyle factors in Model 2, statistically significant associations were observed for RDW (OR = 1.686 [1.171–2.427], *P* = 0.005). Other indicators did not exhibit statistically significant associations with PD prevalence after adjustment.

### Association of inflammation/nutrition-based indicators with mortality

**FIGURE 1:**
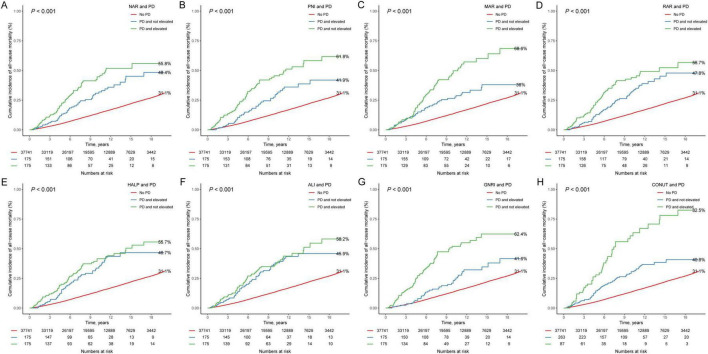
Kaplan-Meier survival curves for elevated inflammation/nutrition-based indicators (**A**: NAR; **B**: PNI; **C**: MAR; **D**: RAR; **E**: HALP; **F**: ALI; **G**: GNRI; and **H**: CONUT) and mortality in participants with PD. NAR, neutrophil-albumin ratio; PNI, prognostic nutritional index; MAR, monocyte-albumin ratio; RAR, red cell distribution width-albumin ratio; HALP, hemoglobin, albumin, lymphocyte, and platelet; ALI, advanced lung cancer inflammation index; GNRI, geriatric nutrition risk index; CONUT, controlling nutritional status.

Over a median follow-up period of 9.33 years (IQR: 5.08–13.83 years), a total of 5776 all-cause deaths were recorded. Kaplan-Meier survival curves for inflammation/nutrition-based indicators and mortality are presented in Figures 1A–H. The results show that participants in the elevated NAR, MAR, RAR, ALI, and CONUT score groups had higher mortality rates, and those in the non-elevated PNI, HALP score, and GNRI groups had higher mortality (all log-rank *P* < 0.001). Table 3 presents the findings from COX regression analysis investigating the association between inflammation/nutrition-based indicators and mortality. In the crude model, all inflammation/nutrition-based indicators showed significant associations with all-cause mortality. After adjusting for demographic factors (age, sex, and race/ethnicity) in Model 1, these associations remained significant for most indicators. Further adjustment for additional variables related to socioeconomic status, lifestyle factors, health status, and nutritional intake in Model 2 confirmed persistent associations between inflammation/nutrition-based indicators (NAR, PNI, MAR, RAR, HALP score, ALI, GNRI, CONUT score) and all-cause mortality. Notably, higher levels of NAR, MAR, RAR, and CONUT score were consistently associated with increased risk of mortality, while higher levels of PNI, HALP score, ALI, and GNRI were associated with decreased risk of mortality. These results indicate that inflammation and nutritional status play important roles in predicting all-cause mortality among adults. Moreover, Supplementary Figure 2 shows the results of the RCS analysis illustrating the multivariate adjusted dose-response relationship between inflammation/nutrition- based indicators and all-cause mortality. The results showed that PNI, MAR, HALP, ALI, GNRI and CONUT were nonlinearly associated with all-cause mortality. Supplementary Figure 3 and Table 4 presents the results of the relationship between inflammation and nutritional indicators and all-cause mortality among adults.

**TABLE 3:** COX regression analysis of the relationship between inflammation/nutrition-based indicators and all-cause mortality among adults in NHANES 1999–2018.

	Crude		Model 1		Model 2	
	HR (95% CI)	*P*-value	HR (95% CI)	*P*-value	HR (95% CI)	*P*-value
NAR	1.482 (1.376, 1.596)	< 0.001	1.534 (1.434, 1.642)	< 0.001	1.245 (1.163, 1.333)	< 0.001
PNI	0.463 (0.435, 0.493)	< 0.001	0.738 (0.692, 0.787)	< 0.001	0.723 (0.674, 0.775)	< 0.001
MAR	1.796 (1.660, 1.942)	< 0.001	1.495 (1.387, 1.610)	< 0.001	1.273 (1.184, 1.369)	< 0.001
RAR	2.846 (2.670, 3.033)	< 0.001	1.930 (1.806, 2.063)	< 0.001	1.653 (1.549, 1.765)	< 0.001
HALP score	0.774 (0.717, 0.835)	< 0.001	0.899 (0.837, 0.965)	0.003	0.841 (0.777, 0.910)	< 0.001
ALI	1.499 (1.402, 1.601)	< 0.001	1.275 (1.198, 1.358)	< 0.001	1.165 (1.092, 1.244)	< 0.001
GNRI	0.838 (0.785, 0.894)	< 0.001	0.818 (0.771, 0.868)	< 0.001	0.753 (0.710, 0.798)	< 0.001
CONUT score	1.396 (1.299, 1.500)	< 0.001	1.343 (1.255, 1.438)	< 0.001	1.312 (1.226, 1.403)	< 0.001

NAR, neutrophil-albumin ratio; PNI, prognostic nutritional index; MAR, monocyte-albumin ratio; RAR, red cell distribution width-albumin ratio; HALP, hemoglobin, albumin, lymphocyte, and platelet; ALI, advanced lung cancer inflammation index; GNRI, geriatric nutrition risk index; CONUT, controlling nutritional status. Inflammation/nutrition-based indicators were classified into two groups based on the median, with participants below the median used as the reference in the regression model. Model 1: Adjusted for age (20–39, 40–59, or ≥ 60 years), sex (male or female), and race/ethnicity (non-Hispanic White, non-Hispanic Black or other race); Model 2: Model 1 + marital status (married/living with partner, or single/divorced/widowed), education level (below high school, high school, or above high school), family PIR ( ≤ 1.0, 1.1–3.0, or > 3.0), drinking status (nondrinker, former drinker, or current drinker), smoking status (never smoker, former smoker, or current smoker), physical activity (inactive, insufficiently active, or active), HEI (in quartiles), and CCI (continous).

**TABLE 4:** COX regression analysis of inflammation/nutrition-based indicators and Parkinson’s disease with all-cause mortality among adults in NHANES 1999–2018.

	Crude		Model 1		Model 2	
	HR (95% CI)	*P*-value	HR (95% CI)	*P*-value	HR (95% CI)	*P*-value
**Parkinson’s disease**						
No	1 [Reference]		1 [Reference]		1 [Reference]	
Yes	3.182 (2.445, 4.142)	< 0.001	2.226 (1.754, 2.825)	< 0.001	1.747 (1.363, 2.238)	< 0.001
**NAR and PD**						
No PD	1 [Reference]		1 [Reference]		1 [Reference]	
PD and not elevated	2.629 (1.811, 3.817)	< 0.001	1.751 (1.251, 2.451)	0.001	1.467 (1.052, 2.046)	0.024
PD and elevated	3.837 (2.620, 5.620)	< 0.001	2.854 (1.986, 4.102)	< 0.001	2.066 (1.398, 3.052)	< 0.001
**PNI and PD**						
No PD	1 [Reference]		1 [Reference]		1 [Reference]	
PD and elevated	2.715 (1.852, 3.980)	< 0.001	2.297 (1.560, 3.382)	< 0.001	1.715 (1.137, 2.586)	0.010
PD and not elevated	3.658 (2.628, 5.092)	< 0.001	2.175 (1.612, 2.935)	< 0.001	1.771 (1.295, 2.423)	< 0.001
**MAR and PD**						
No PD	1 [Reference]		1 [Reference]		1 [Reference]	
PD and not elevated	2.025 (1.350, 3.038)	< 0.001	1.695 (1.135, 2.532)	0.01	1.308 (0.862, 1.984)	0.207
PD and elevated	5.143 (3.660, 7.227)	< 0.001	2.815 (2.058, 3.850)	< 0.001	2.249 (1.612, 3.138)	< 0.001
**RAR and PD**						
No PD	1 [Reference]		1 [Reference]		1 [Reference]	
PD and not elevated	2.789 (1.953, 3.983)	< 0.001	2.198 (1.552, 3.114)	< 0.001	1.898 (1.299, 2.775)	< 0.001
PD and elevated	3.703 (2.617, 5.240)	< 0.001	2.255 (1.680, 3.027)	< 0.001	1.617 (1.179, 2.218)	0.003
**HALP and PD**						
No PD	1 [Reference]		1 [Reference]		1 [Reference]	
PD and elevated	3.096 (2.159, 4.438)	< 0.001	2.538 (1.851, 3.481)	< 0.001	1.757 (1.249, 2.470)	0.001
PD and not elevated	3.262 (2.346, 4.536)	< 0.001	2.011 (1.466, 2.758)	< 0.001	1.738 (1.242, 2.432)	0.001
**ALI and PD**						
No PD	1 [Reference]		1 [Reference]		1 [Reference]	
PD and not elevated	2.799 (1.888, 4.148)	< 0.001	2.142 (1.532, 2.993)	< 0.001	1.726 (1.244, 2.394)	0.001
PD and elevated	3.571 (2.503, 5.096)	< 0.001	2.298 (1.666, 3.171)	< 0.001	1.763 (1.225, 2.537)	0.002
**CONUT and PD**						
No PD	1 [Reference]		1 [Reference]		1 [Reference]	
PD and not elevated	2.512 (1.775, 3.556)	< 0.001	2.149 (1.581, 2.921)	< 0.001	1.570 (1.149, 2.146)	0.005
PD and elevated	6.446 (4.468, 9.301)	< 0.001	2.388 (1.577, 3.618)	< 0.001	2.221 (1.434, 3.440)	< 0.001
**GNRI and PD**						
No PD	1 [Reference]		1 [Reference]		1 [Reference]	
PD and elevated	1.921 (1.249, 2.954)	0.003	1.416 (0.995, 2.015)	0.053	1.013 (0.696, 1.474)	0.947
PD and not elevated	4.671 (3.291, 6.629)	< 0.001	3.082 (2.244, 4.233)	< 0.001	2.689 (1.898, 3.811)	< 0.001

NAR, neutrophil-albumin ratio; PNI, prognostic nutritional index; MAR, monocyte-albumin ratio; RAR, red cell distribution width-albumin ratio; HALP, hemoglobin, albumin, lymphocyte, and platelet; ALI, advanced lung cancer inflammation index; GNRI, geriatric nutrition risk index; CONUT, controlling nutritional status. Model 1: Adjusted for age (40–59, or ≥ 60 years), race/ethnicity (non-Hispanic White, non-Hispanic Black or other race), living status (with partners, or alone), education level (below high school, high school, or above high school), family PIR ( < 1.0, or ≥ 1.0), and BMI ( < 25.0, 25.0–29.9, or > 29.9 kg/m^2^); Model 2: Model 1 + drinking status (nondrinker, low-to-moderate drinker, or heavy drinker), smoking status (never smoker, former smoker, or current smoker), physical activity (inactive, insufficiently active, or active), HEI (in quartiles), and CCI (continuous).

### Prediction of mortality by inflammation/nutrition-based indicators and their components

**FIGURE 2:**
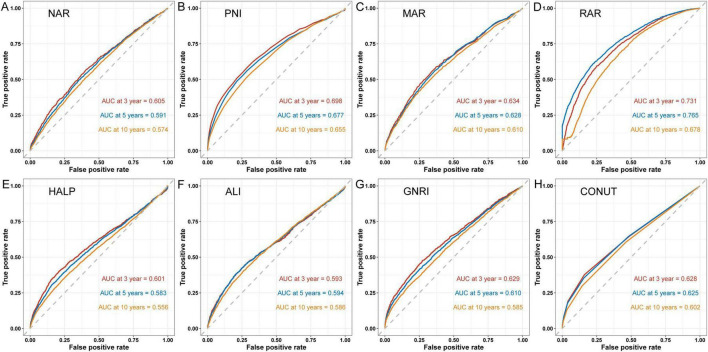
Predictive capability of time-dependent ROC assessment of inflammation/nutrition-based indicators (**A**: NAR; **B**: PNI; **C**: MAR; **D**: RAR; **E**: HALP; **F**: ALI; **G**: GNRI; and **H**: CONUT) for 3-, 5-, and 10-year all-cause mortality. NAR, neutrophil-albumin ratio; PNI, prognostic nutritional index; MAR, monocyte-albumin ratio; RAR, red cell distribution width-albumin ratio; HALP, hemoglobin, albumin, lymphocyte, and platelet; ALI, advanced lung cancer inflammation index; GNRI, geriatric nutrition risk index; CONUT, controlling nutritional status.

**FIGURE 3:**
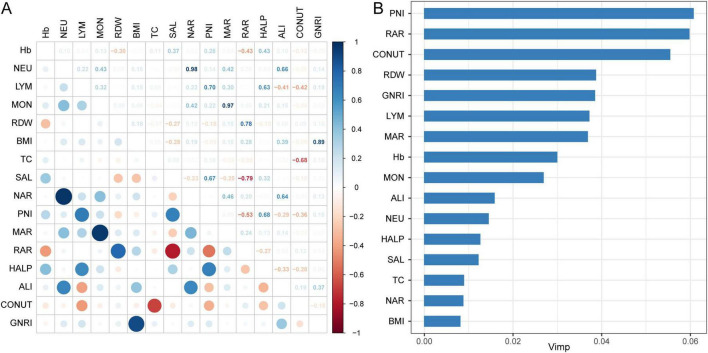
Capability of inflammation/nutrition-based indicators and its components to predict mortality. **(A)** Spearman’s correlation analysis was used to calculate the correlation coefficients among inflammation/nutrition-based indicators and its components; **(B)** Ranking the significance of the inflammation/nutrition-based indicators and its components in predicting all-cause mortality using RSF modeling.

Figure 2 shows time-dependent ROC curves assessing the predictive value of predicting all-cause mortality for inflammation/nutrition-based indicators in adult participants at 3, 5, and 10 years. The results showed that RAR had the highest AUC values of 0.731, 0.765, and 0.678 for 3, 5, and 10 years, respectively, followed by PNI with AUC values of 0.689, 0.677, and 0.655, respectively. Supplementary Figure 4 shows the time-dependent ROC curves for the inflammation and nutritional indicators of all-cause mortality in adult participants. Figure 3A shows the matrix of correlation coefficients based on the correlation between the inflammation/nutrition-based indicators and their components. In addition, the RSF model emphasized the importance of inflammation/nutrition-based indicators and their components in predicting mortality, with PNI and RAR being the most important indicators for predicting all-cause mortality (Figure 3B).

### Association of inflammation/nutrition-based indicators and PD with mortality

The Kaplan-Meier survival curves showed a higher mortality rate for participants in the PD group compared to the non-PD group (Supplementary Figure 5). Table 4 presents the results of COX regression analysis examining the association between inflammation/nutrition-based indicators and PD (PD) with all-cause mortality among adult. The results indicate that individuals with PD have a significantly higher risk of all-cause mortality compared to those without PD, even after adjusting for various demographic and lifestyle factors (HR = 1.747 [1.363–2.238], *P* < 0.001). Moreover, the analysis demonstrates that elevated levels of inflammation/nutrition-based indicators are associated with a higher risk of all-cause mortality among individuals with PD compared to those without PD. This suggested a synergistic effect of PD and elevated levels of inflammatory/nutritional indicators on mortality risk. Specifically, individuals with PD and elevated NAR (HR = 2.066 [1.398–3.052], *P* < 0.001), MAR (HR = 2.249 [1.612–3.138], *P* < 0.001), RAR (HR = 1.617 [1.179–2.218], *P* 0.003), ALI (HR = 1.763 [1.225–2.537], *P* = 0.002) and CONUT (HR = 2.221 [1.434–3.440], *P* < 0.001), and not elevated PNI (HR = 1.771 [1.295–2.423], *P* < 0.001), HALP (HR = 1.738 [1.242–2.432], *P* = 0.001), and GNRI (HR = 2.689 [1.898–3.811], *P* < 0.001) have a substantially higher risk of mortality compared to those without PD.

## Discussion

### Summary of findings

In our study, we discussed the associations between various inflammation/nutrition-based indicators and mortality among adult population and individuals with PD from NHANES. It suggested that certain inflammation/nutrition-based indicators such as PNI, RAR, RDW, NAR, MAR, ALI, CONUT score, HALP score, and GNRI were associated with mortality rates in adults. Higher levels of NAR, MAR, RAR, and CONUT score were linked with increased mortality rates, while higher levels of PNI, HALP score, ALI, and GNRI are associated with decreased mortality risk. The RCS result indicated that nonlinear associations between these indicators and mortality. The random survival forest emphasized the significance of inflammation/nutrition-based indicators in predicting mortality, with PNI and RAR being particularly important predictors of all-cause mortality. Elevated levels of inflammation/nutrition-based indicators seemed to increase this risk, suggesting a synergistic effect between PD and these indicators on mortality risk. Overall, our findings underscored the importance of monitoring and managing nutritional and inflammatory status in individuals with PD to potentially reduce mortality risk.

### Importance of inflammation and nutrition in PD

Nutrition status and inflammation has emerged as a crucial factor in slowing down the progression of numerous chronic diseases. In a prospective study, PD occurred in older individuals and increased the risk of mortality (27). For patients with PD, the survival outcomes are significantly influenced by their immunological status, which also includes factors, nutritional health and inflammatory responses. Current biomarkers for PD have primarily focused on detecting disease and evaluating symptoms. Limited studies have been conducted to explore the prognostic value of novel biomarkers in PD. Previous research has indicated that motor deterioration and prognosis in PD may be linked to elevated levels of serum CRP (28). Gao et al. (14) explored the role of C-reactive protein-albumin ratio on the outcome of survival and all-cause death based on data from 235 PD patients (14). They found higher level of C-reactive protein-albumin ratio was associated with the all-cause mortality in the PD, which showed better predictive value compared to its component CRP (14). Another study based on Japan patients with PD suggested that elevated serum albumin levels may have a protective effect against severe motor impairment and PD-related death (12). The above evidence supported the roles of inflammation and nutrition in the pathogenesis of PD. However, most of studies only investigated a single serum biomarker and did not delve deeper into the relationship between combinations of biomarkers and PD. We worked on evaluating the inflammation/nutrition-based biomarkers to follow the prognosis of the PD. The results of this research underscored the significance of inflammation/nutrition-based indicators in forecasting mortality, and the PNI and RAR showed the best predictive value for predicting all-cause mortality. It’s worth noting that PD and elevated levels of inflammatory/nutritional indicators behaved a synergistic effect on mortality risk.

### Prognostic Nutritional Index (PNI)

The variables of PNI values include serum albumin and peripheral blood lymphocyte counts. As a multifunctional protein, serum albumin levels can to some extent reflect the body’s protein reserves and have significant neuroprotective effects against PD though modulating intracellular signaling in neuronal, glial cells, its antioxidant properties and inhibiting polymerization (29). Also, alterations in serum albumin levels might influence the accumulation of peptide amyloid beta (Aβ) plaques (29). Recent studies showed there was an inverse association between serum albumin level and Aβ deposition as well as Aβ positivity (30). The level of lymphocytes serves as an indicator of the individual’s immune system function. A decrease in lymphocyte levels may indicate immune suppression or an underlying health condition, while an increase may suggest an active immune response to an infection or inflammation (31). PNI, initially introduced by Smale et al., primarily assesses the likelihood of cancer recurrence and survival post-surgery (32). PNI has often been used as part of cancer staging to evaluate the survival outcomes of cancer spread in various types of cancer (33, 34). It’s also reported that PNI may serve as a predictor of long-term mortality in older adults who have had an ischemic stroke (35). In recent years, a growing body of research has supported the utilization of PNI assessment for prognostic purposes in patients with poor cognitive performance (36). The PNI has shown promise as a reliable predictor of the odd of cognitive function impairment in the elderly population (36). However, the application of PNI in predicting the risk of mortality in PD has been relatively unexplored. In our study, The AUC values for PNI were around 0.655–0.689 in participants at 3, 5, and 10 years, which showed better predictive value of predicting all-cause mortality in adult participants.

### Red cell distribution width-albumin ratio (RAR)

The RAR is an innovative inflammatory biomarker that combines two distinct blood parameters: red cell distribution width (RDW) and albumin levels (37). RDW levels are employed in evaluating systemic inflammation, which indicates the variation in the size of red blood cells circulating in the bloodstream (38). Albumin is a protein produced by the liver that plays a crucial role in maintaining blood volume and regulating various functions. The primary application of the RAR in evaluating outcomes in patients with solid stroke and acute respiratory distress syndrome highlighted its potential as a reliable indicator of inflammation (39, 40). Recently, elevated RDW levels have been associated with poorer cognitive performance and an increased risk of dementia in several studies (41). Research suggested that inflammation and oxidative stress may contribute to neurodegenerative processes, leading to cognitive impairment and ultimately dementia (42). There are currently limited studies demonstrating a correlation between RDW or RAR and the prognosis of PD patients. A recent study examined the correlation between RDW levels and disease severity in 94 patients with PD (43). They found, in PD patients, RDW levels are elevated compared to healthy individuals, but there is no correlation observed between RDW levels and disease development (43). We proved that RAR was associated with the prevalence of PD among adult. Besides, RAR had the highest AUC values for predicting outcomes at long term follow-up in adult participants.

### Other inflammation/nutrition-based indicators

In addition to PNI and RAR, other inflammation/nutrition-based indicators, including NAR, MAR, ALI, CONUT score, HALP score, and GNRI, were also associated with all-cause mortality. These inflammation/nutrition-based biomarkers, in conjunction with PD, collectively increase the risk of all-cause mortality. The HALP score has been identified as a readily accessible metric for assessing both systemic inflammation and nutritional status in patients with multiple malignancies (44). A recent study found that the lower HALP scores were significantly associated with a heightened risk of cognitive impairment within a two-week timeframe among patients with acute ischemic stroke (45). CONUT consists of three components: nutritional indicators such as serum albumin, along with immune status indicators including cholesterol and lymphocyte count. It’s plausible to consider employing the CONUT score as a promising prognostic marker for screening the risk of mortality or impaired physical function among patients in stoke (46). Other inflammation indicators (NAR, ALI, GNRI) have been suggested to be associated with different type of health hazards in adults, including neurodegenerative disorders (20, 47, 48). One of potential mechanisms is that inflammatory indicators can breach the blood-brain barrier and can trigger inflammatory responses within the brain, leading to damage to nerve cells and contributing to neurodegeneration (49). Chronic inflammation is often observed in affected brain regions, and there’s evidence suggesting that targeting inflammation could potentially slow down disease progression (50). Assessing both nutrition and serum inflammation levels can provide valuable insights into early steps to develop management plan and predict outcomes of PD patients.

### Study strengths

As we know, this is one of most comprehensive studies that analyzed the roles of several common inflammation/nutrition-based indicators in prognosis of PD patients or adults. Previous studies may focus on one or two indicators in peripheral blood or cerebrospinal fluid. We demonstrated that inflammation/nutrition-based indicators were significantly related with all-cause mortality, especially PNI and RAR performed better predictive ability in predicting the risk of mortality. Unlike some other screening biomarkers that might require more complex or specialized tests, these indicators often rely on straightforward laboratory parameters. This study will provide more evidence for clinical doctors to remind them that controlling systemic inflammation and offering enough nutrition is significant for individuals, especially for individuals with PD.

### Study limitations

Despite our findings, several limitations must be acknowledged. Firstly, the results of our study only demonstrated some relationships between certain inflammation/nutrition-based indicators with PD and mortality, but could not suggest inflammatory response or nutritional condition play causal roles in PD or mortality. Specific causal associations need more longitudinal studies to prove, such as Mendelian randomization. Furthermore, PD is defined by self-reported use of medication specifically indicated for treating PD. Some individuals had the possibility to be unaware of their PD symptoms in the NHANES, that may increase the potential for bias in the study. In addition, our study is the reliance on a single baseline measurement of inflammation/nutrition-based indicators. This approach may not fully capture the dynamic nature of these indicators, as their levels can fluctuate over time. Changes in these indicators throughout the study period might have a more pronounced relationship with mortality risk than a single snapshot measurement. Longitudinal tracking of these indicators could offer a more comprehensive understanding of their temporal variations and their evolving impact on mortality risk. Future studies employing repeated measures of these biomarkers over time could provide valuable insights into their dynamic relationship with patient outcomes and potentially reveal more accurate associations with mortality.

## Conclusion

This study examined the association between inflammation and nutrition-based indicators and their impact on mortality risk among adults, with a particular focus on PD. Utilizing data from NHANES 1999–2018, our analysis identified a significant interaction between elevated levels of these indicators and PD in relation to all-cause mortality. These findings underscore the importance of considering both inflammatory and nutritional status when assessing mortality risk in PD patients. Moreover, the results highlight the need for future research to explore the mechanisms underlying these relationships and to investigate the potential for targeted interventions to mitigate mortality risk. Overall, this study contributes to a deeper understanding of how inflammation and nutrition influence mortality outcomes in the context of PD and emphasizes the need for ongoing research in this area to inform clinical practice.

## Data Availability

The original contributions presented in the study are included in the article/Supplementary material, further inquiries can be directed to the corresponding author.
