# Supplementary material for: Association of inflammation/nutrition-based indicators with Parkinson’s disease and mortality
Source: Front Nutr. 2024 Sep 17;11:1439803. doi: 10.3389/fnut.2024.1439803 (PMC11442420; doi:10.3389/fnut.2024.1439803)
Supplement: Supplementary file 1 [file Data_Sheet_1.pdf]

Online Supplementary Material

Association of inflammation/nutrition-based indicators with Parkinson's disease and Mortality

Figure S1. Flowchart of the study

Table S1. Calculation methods of combination in each nutrition/inflammation-based indicator.

Table S2. Baseline characteristics of adult participants according to Parkinson's disease in NHANES 1999–2018.

Table S3. Logistic regression analysis of the relationship between inflammation and nutritional indicators and the prevalence of Parkinson's disease among adults in NHANES 1999–2018.

Table S4. COX regression analysis of the relationship between inflammation and nutritional indicators and all-cause mortality among adults in NHANES 1999–2018.

Figure S2. Kaplan-Meier survival curves for inflammation/nutrition-based indicator and mortality in adults.

Figure S3. Restricted cubic spline (RCS) analysis with multivariate-adjusted associations of inflammation/nutrition-based indicators wih all-cause mortality among adults in NHANES 1999–2018.

Figure S4. Predictive value of time-dependent ROC assessment of inflammation and nutritional indicator for 3-, 5-, and 10-year all-cause mortality.

Figure S5. Kaplan-Meier survival curves for Parkinson's disease and mortality in adults.

Materials and methods: Other Covariates

This section provides a comprehensive definition of potential confounding variables including family poverty income ratio (PIR, ≤1.0, 1.1–3.0, or >3.0), smoking status (never, former, or current smoker), drinking status (nondrinker, former drinker, or current drinker), physical activity (inactive, insufficiently active, or active), healthy eating index (HEI), and Charlson comorbidity index (CCI).

Family poverty income ratio Income was assessed using the poverty income ratio (PIR, the ratio of family income divided by a poverty threshold specific for family size using guidelines from the US Department of Health and Human Services) and categorized as ≤1.0, 1.1-3.0 and >3.0 [1].

Smoking status Never smokers were classified as those who reported smoking <100 cigarettes during their lifetime. Those who smoked >100 cigarettes in their lifetime were considered as current smokers, and those who smoked >100 cigarettes and had quit smoking were considered as former smokers [2].

Drinking status Drinking status was classified as nondrinker, low-to-moderate drinker (<2 drinks/day in men and <1 drink/day in women), or heavy drinker (≥2 drinks/day in men and ≥1 drinks/day in women) [2].

Physical activity Physical activity was categorized as inactive group (no leisure-time physical activity), insufficiently active group (leisure time moderate activity 1–5 times per week with MET ranging from 3 to 6 or leisure-time vigorous activity 1–3 times per week with MET >6), or active group (those who had more leisure-time moderate-or-vigorous activity than above) [3].

Healthy Eating Index The Healthy Eating Index (HEI) is a measure calculated from 24-hour dietary recall data to assess diet quality based on the 2015–2020 Dietary Guidelines for Americans (DGA) [4]. It comprises 13 subgroups, with a total possible score of 100. Nine components evaluate adequacy (higher intakes contribute to a higher score) including total fruits, whole fruits, total vegetables, greens and beans, whole grains, dairy, total protein foods, seafood and plant proteins, and fatty acids. The remaining four components assess moderation (lower intakes yield a higher score), covering refined grains, sodium, added sugars, and saturated fats. The HEI offers a comprehensive

framework for understanding dietary patterns and their relationship to health outcomes, with scores reflecting adherence to key dietary recommendations.

Charlson Comorbidity Index The Charlson Comorbidity Index (CCI) is a method used to quantify an individual's overall health status by summing the scores assigned to various diseases [5]. It assumes respondents without reported diseases as healthy, assigning a zero value to unreported conditions. This scoring approach follows the methodology established by Zhao et al. in prior research [6], ensuring consistency in the assessment of comorbidities.

References

1. **Services USDoHaH. Poverty Guidelines, Research, and Measurement** [http://aspe.hhs.gov/POVERTY/index.shtml.]
2. Qiu Z, Chen X, Geng T, Wan Z, Lu Q, Li L, Zhu K, Zhang X, Liu Y, Lin X *et al*: **Associations of Serum Carotenoids With Risk of Cardiovascular Mortality Among Individuals With Type 2 Diabetes: Results From NHANES**. *Diabetes Care* 2022, **45**(6):1453-1461.
3. Beddhu S, Baird BC, Zitterkoph J, Neilson J, Greene T: **Physical activity and mortality in chronic kidney disease (NHANES III)**. *Clin J Am Soc Nephrol* 2009, **4**(12):1901-1906.
4. Kirkpatrick SI, Reedy J, Krebs-Smith SM, Pannucci TE, Subar AF, Wilson MM, Lerman JL, Tooze JA: **Applications of the Healthy Eating Index for Surveillance, Epidemiology, and Intervention Research: Considerations and Caveats**. *J Acad Nutr Diet* 2018, **118**(9):1603-1621.
5. Kim CY, Sivasundaram L, LaBelle MW, Trivedi NN, Liu RW, Gillespie RJ: **Predicting adverse events, length of stay, and discharge disposition following shoulder arthroplasty: a comparison of the Elixhauser Comorbidity Measure and Charlson Comorbidity Index**. *J Shoulder Elbow Surg* 2018, **27**(10):1748-1755.
6. Zhao H, Pan Y, Wang C, Guo Y, Yao N, Wang H, Li B: **The Effects of Metal Exposures on Charlson Comorbidity Index Using Zero-Inflated Negative Binomial Regression Model: NHANES 2011-2016**. *Biol Trace Elem Res* 2021, **199**(6):2104-2111.

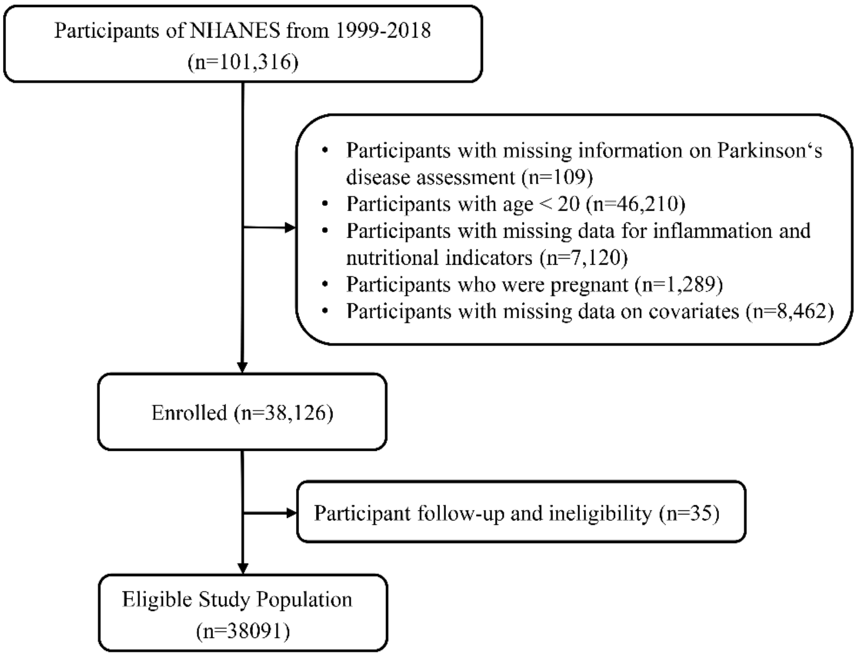

Figure S1. Flowchart of the study

Table S1. Calculation methods of combination in each nutrition/inflammation-based indicator.

Indicators	Definition or calculation formula				
NAR	neutrophil count ($\times 10^9$)/ albumin (g/ L)				
PNI	albumin (g/L) + 5×lymphocyte count ($\times 10^9$)				
MAR	monocyte count ($\times 10^9$)/ albumin (g/ L)				
RAR	RDW (%)/ albumin (g/ L)				
HALP score	[hemoglobin (g/L) × albumin (g/L) × lymphocytes ($\times 10^9$)]/platelets ($\times 10^9$)				
ALI	BMI (kg/m^2) × albumin(g/dl)/NLR ($\times 10^9$)				
GNRI	1.489×albumin (g/L) + (41.7× current weight/ ideal body weight (IBW)				
CONUT score	albumin score+ TLC score+ T-cho score				
Serum albumin (g/dL)	albumin score	Total lymphocyte (count/mm^3)	TLC score	Total cholesterol (mg/dL)	T-cho score
3.5-4.5	1	≥1600	0	≥180	0
30-34.9	2	1200-1599	1	140-180	1
25-29.9	4	800-1199	2	100-139	2
<25	6	800	3	100	3

If current weight is greater than IBW, current/IBW is regarded as 1. The IBW of men and women were calculated by the formula, height(cm)-100-[height (cm)-150/4] and height(cm)-100-[height (cm)-150/2.5], respectively.

Table S2. Baseline characteristics of adult participants according to Parkinson's disease in NHANES 1999–2018.

Characteristics	Total	Parkinson's disease		*P* value
		No (n=37741)	Yes (n=350)	
Age, %				<0.001
20-39 years	12420(36.62)	12383(36.82)	37(12.40)	
40-59 years	12519(38.43)	12396(38.37)	123(46.39)	
≥60 years	13152(24.95)	12962(24.81)	190(41.21)	
Sex, %				0.014
Female	18869(50.52)	18693(50.46)	176(58.42)	
Male	19222(49.48)	19048(49.54)	174(41.58)	
Race/ethnicity, %				<0.001
Non-Hispanic White	18033(71.13)	17810(71.05)	223(81.09)	
Non-Hispanic Black	7505(9.93)	7455(9.95)	50(7.77)	
Other race	12553(18.94)	12476(19.00)	77(11.14)	
Marital status, %				0.377
Married/living with partner	14974(35.56)	14820(35.53)	154(38.69)	
Single/divorced/widowed	23117(64.44)	22921(64.47)	196(61.31)	
Education level, %				0.251
Below high school	9572(15.84)	9464(15.81)	108(20.12)	
High school	8911(24.18)	8840(24.18)	71(23.91)	
Above high school	19608(59.98)	19437(60.02)	171(55.98)	
Family PIR, %				< 0.001
≤1.0	7470(13.38)	7385(13.32)	85(20.56)	
1.1–3.0	16048(35.76)	15884(35.71)	164(41.39)	

>3.0	14573(50.86)	14472(50.97)	101(38.05)	
Smoking status, %				0.445
Never smoker	20236(53.26)	20071(53.28)	165(52.50)	
Former smoker	9793(25.31)	9689(25.34)	104(23.01)	
Current smoker	8043(21.40)	7962(21.38)	81(24.49)	
Drinking status, %				0.076
Nondrinker	8546(18.24)	8448(18.19)	98(24.45)	
Low-to-moderate drinker	26263(71.68)	26031(71.71)	232(67.58)	
Heavy drinker	3282(10.08)	3262(10.10)	20(7.97)	
Physical activity, %				<0.001
Inactive	9934(20.99)	9795(20.88)	139(33.93)	
Insufficiently active	14405(40.78)	14296(40.83)	109(34.41)	
Active	13752(38.23)	13650(38.29)	102(31.67)	
HEI-2015 score	49.53(40.47,59.37)	49.53(40.48,59.38)	49.72(39.85,57.02)	0.435
CCI	0.86(0.01)	0.85(0.01)	1.81(0.13)	<0.001
Inflammation and nutritional indicators				
Neutrophil, $10^3/\mu L$	4.00(3.20,5.10)	4.00(3.20,5.10)	4.30(3.10,5.30)	0.154
Lymphocyte, $10^3/\mu L$	2.00(1.60,2.50)	2.00(1.60,2.50)	1.90(1.50,2.50)	0.044
Monocyte, $10^3/\mu L$	0.50(0.40,0.70)	0.50(0.40,0.70)	0.50(0.40,0.70)	0.761
Hemoglobin, g/dL	14.40(13.40,15.40)	14.40(13.40,15.40)	14.00(13.10,15.00)	<0.001
RDW, %	12.80(12.30,13.50)	12.80(12.30,13.50)	13.20(12.70,14.00)	<0.001
Serum albumin, g/L	43.00(41.00,45.00)	43.00(41.00,45.00)	41.00(40.00,44.00)	<0.001
Total cholesterol, mg/dL	193.00(167.00,221.00)	193.00(167.00,221.00)	196.00(166.00,225.00)	0.501
Body mass index, kg/m^2	27.71(24.10,32.22)	27.70(24.10,32.20)	29.20(24.40,33.89)	0.010
Inflammation/nutrition-based indicators				

NAR	0.09(0.07,0.12)	0.09(0.07,0.12)	0.10(0.08,0.13)	0.016
PNI	53.50(50.50,56.50)	53.50(50.50,56.50)	51.50(49.00,55.00)	<0.001
MAR	0.01(0.01,0.02)	0.01(0.01,0.02)	0.01(0.01,0.02)	0.358
RAR	5.73(4.83,6.86)	5.73(4.83,6.86)	5.73(4.76,7.15)	0.819
HALP score	0.30(0.28,0.32)	0.30(0.28,0.32)	0.32(0.30,0.34)	<0.001
ALI	50.57(39.00,65.49)	50.60(39.03,65.55)	47.20(34.45,60.97)	0.001
GNRI	120.18(113.30,128.97)	120.17(113.30,128.95)	121.33(113.07,133.20)	0.191
CONUT score	0.00(0.00,1.00)	0.00(0.00,1.00)	1.00(0.00,1.00)	0.247

Abbreviations: HEI-2015, Healthy Eating Index 2015; CCI, Charlson Comorbidity Index; RDW, red cell distribution width; NAR, neutrophil-albumin ratio; PNI, prognostic nutritional index; MAR, monocyte-albumin ratio; RAR, red cell distribution width-albumin ratio; HALP, hemoglobin, albumin, lymphocyte, and platelet; ALI, advanced lung cancer inflammation index; GNRI, geriatric nutrition risk index; CONUT, controlling nutritional status.

Normally distributed continuous variables are described as means ± SEs, and continuous variables without a normal distribution are presented as medians [interquartile ranges]. Categorical variables are presented as numbers (percentages). N reflect the study sample while percentages reflect the survey-weighted data.

Table S3. Logistic regression analysis of the relationship between inflammation and nutritional indicators and the prevalence of Parkinson's disease among adults in NHANES 1999–2018.

	Crude		Model 1		Model 2	
	OR (95% CI)	*P* value	OR (95% CI)	*P* value	OR (95% CI)	*P* value
NEU	1.293(0.989,1.692)	0.061	1.304(0.991,1.715)	0.058	1.109(0.826,1.487)	0.489
LYM	0.779(0.587,1.034)	0.083	0.910(0.690,1.200)	0.502	0.829(0.618,1.112)	0.209
MON	0.950(0.727,1.242)	0.706	0.929(0.708,1.220)	0.595	0.833(0.631,1.100)	0.195
Hb	0.643(0.475,0.871)	0.005	0.705(0.487,1.022)	0.065	0.709(0.492,1.021)	0.064
RDW	2.261(1.609,3.179)	<0.001	1.983(1.397,2.816)	<0.001	1.686(1.171,2.427)	0.005
SAL	0.565(0.403,0.794)	0.001	0.704(0.496,0.999)	0.050	0.797(0.562,1.129)	0.200
TC	1.094(0.830,1.441)	0.523	0.884(0.664,1.177)	0.397	0.971(0.733,1.287)	0.837
BMI	1.344(1.015,1.779)	0.039	1.244(0.941,1.645)	0.124	1.098(0.838,1.437)	0.495

Abbreviations: NEU, neutrophil; LYM, lymphocyte; MON, monocyte; Hb, hemoglobin; RDW, red cell distribution width; SAL, serum albumin; TC, total cholesterol; BMI, body mass index; Data are presented as OR (95% CI) unless indicated otherwise. Inflammation/nutrition-based indicators were classified into two groups based on the median, with participants below the median used as the reference in the regression model.

Model 1: Adjusted for age (20-39, 40-59, or ≥60 years), sex (male or female), and race/ethnicity (non-Hispanic White, non-Hispanic Black or other race); Model 2: Model 1 + marital status (married/living with partner, or single/divorced/widowed), education level (below high school, high school, or above high school), family PIR (≤1.0, 1.1–3.0, or >3.0), drinking status (nondrinker, former drinker, or current drinker), smoking status (never smoker, former smoker, or current smoker), physical activity (inactive, insufficiently active, or active), HEI (in quartiles), and CCI (continous).

Table S4. COX regression analysis of the relationship between inflammation and nutritional indicators and all-cause mortality among adults in NHANES 1999–2018.

	Crude		Model 1		Model 2	
	HR (95% CI)	*P* value	HR (95% CI)	*P* value	HR (95% CI)	*P* value
NEU	1.270(1.186,1.359)	<0.001	1.386(1.303, 1.476)	<0.001	1.137(1.064, 1.215)	<0.001
LYM	0.684(0.641,0.729)	<0.001	0.929(0.872, 0.990)	0.024	0.827(0.778, 0.880)	<0.001
MON	1.591(1.473,1.719)	<0.001	1.415(1.315, 1.522)	<0.001	1.214(1.131, 1.304)	<0.001
Hb	0.729(0.684,0.778)	<0.001	0.701(0.646, 0.760)	<0.001	0.692(0.638, 0.750)	<0.001
RDW	2.782(2.594,2.982)	<0.001	1.928(1.793, 2.072)	<0.001	1.650(1.534, 1.775)	<0.001
SAL	0.475(0.444,0.508)	<0.001	0.663(0.620, 0.708)	<0.001	0.713(0.666, 0.764)	<0.001
TC	0.990(0.930,1.053)	0.744	0.810(0.766, 0.858)	<0.001	0.874(0.824, 0.927)	<0.001
BMI	1.110(1.036,1.189)	0.003	0.929(0.872, 0.991)	0.024	0.849(0.798, 0.904)	<0.001

Abbreviations: NEU, neutrophil; LYM, lymphocyte; MON, monocyte; Hb, hemoglobin; RDW, red cell distribution width; SAL, serum albumin; TC, total cholesterol; BMI, body mass index; Data are presented as OR (95% CI) unless indicated otherwise. Inflammation/nutrition-based indicators were classified into two groups based on the median, with participants below the median used as the reference in the regression model.

Model 1: Adjusted for age (20-39, 40-59, or ≥60 years), sex (male or female), and race/ethnicity (non-Hispanic White, non-Hispanic Black or other race); Model 2: Model 1 + marital status (married/living with partner, or single/divorced/widowed), education level (below high school, high school, or above high school), family PIR (≤1.0, 1.1–3.0, or >3.0), drinking status (nondrinker, former drinker, or current drinker), smoking status (never smoker, former smoker, or current smoker), physical activity (inactive, insufficiently active, or active), HEI (in quartiles), and CCI (continous).

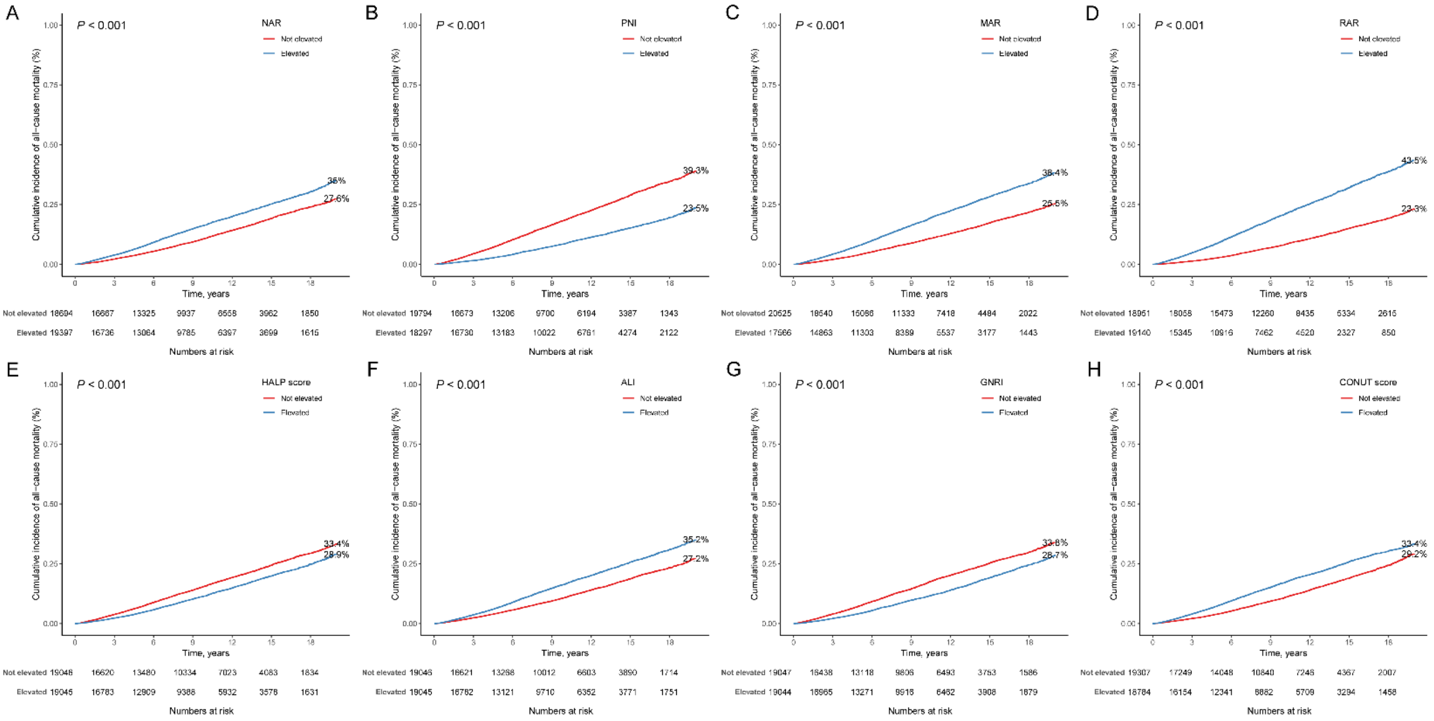

Figure S2. Kaplan-Meier survival curves for inflammation/nutrition-based indicators (A: NAR; B: PNI; C: MAR; D: RAR; E: HALP; F: ALI; G: GNRI; and H: CONUT) and mortality in adults. NAR, neutrophil-albumin ratio; PNI, prognostic nutritional index; MAR, monocyte-albumin ratio; RAR, red cell distribution width-albumin ratio; HALP, hemoglobin, albumin, lymphocyte, and platelet; ALI, advanced lung cancer inflammation index; GNRI, geriatric nutrition risk index; CONUT, controlling nutritional status.

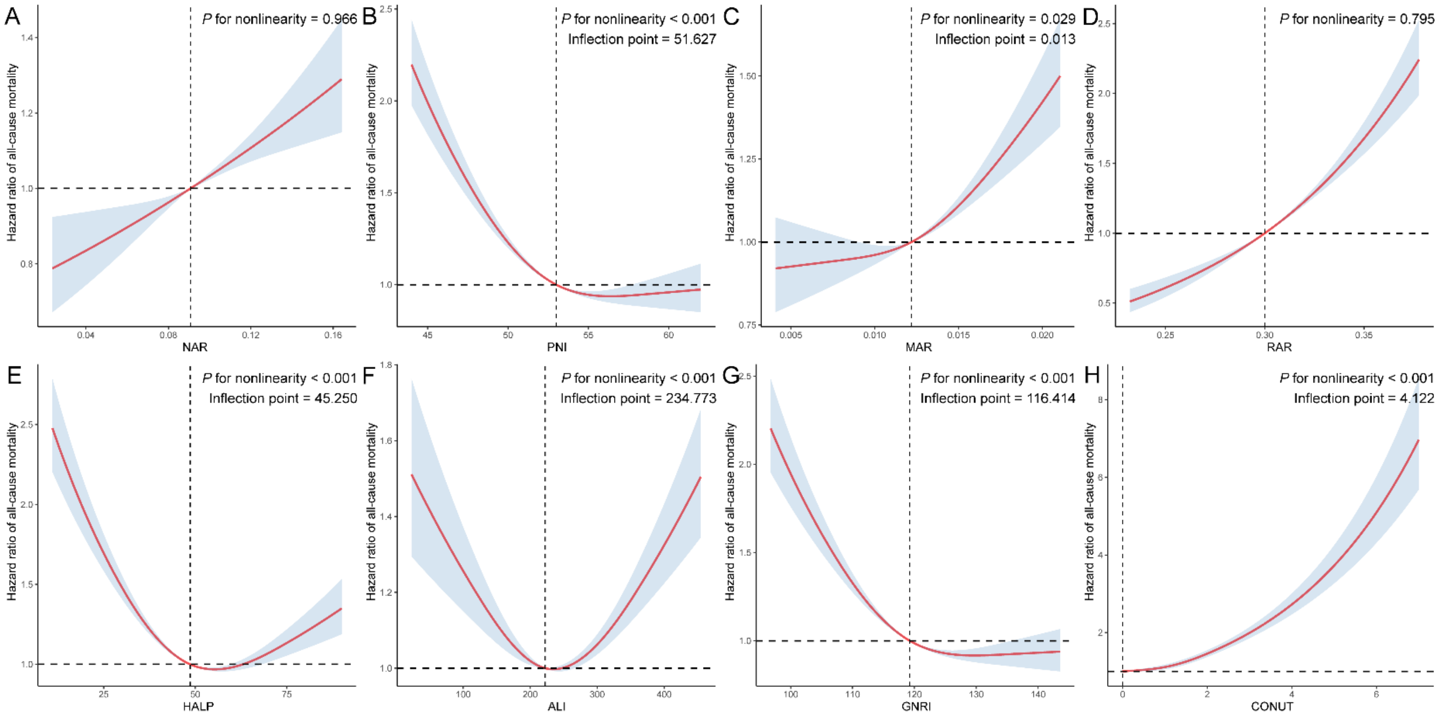

Figure S3. Restricted cubic spline (RCS) analysis with multivariate-adjusted associations of inflammation/nutrition-based indicators (A: NAR; B: PNI; C: MAR; D: RAR; E: HALP; F: ALI; G: GNRI; and H: CONUT) wih all-cause mortality among adults in NHANES 1999–2018. Models are adjusted for age (20-39, 40-59, or ≥60 years), sex (male or female), race/ethnicity (non-Hispanic White, non-Hispanic Black or other race), marital status (married/living with partner, or single/divorced/widowed), education level (below high school, high school, or above high school), family PIR (≤1.0, 1.1–3.0, or >3.0), drinking status (nondrinker, former drinker, or current drinker), smoking status (never smoker, former smoker, or current smoker), physical activity (inactive, insufficiently active, or active), HEI (in quartiles), and CCI (continous). NAR, neutrophil-albumin ratio; PNI, prognostic nutritional index; MAR, monocyte-albumin ratio; RAR, red cell distribution width-albumin ratio; HALP, hemoglobin, albumin, lymphocyte, and platelet; ALI, advanced lung cancer inflammation index; GNRI, geriatric nutrition risk index; CONUT, controlling nutritional status.

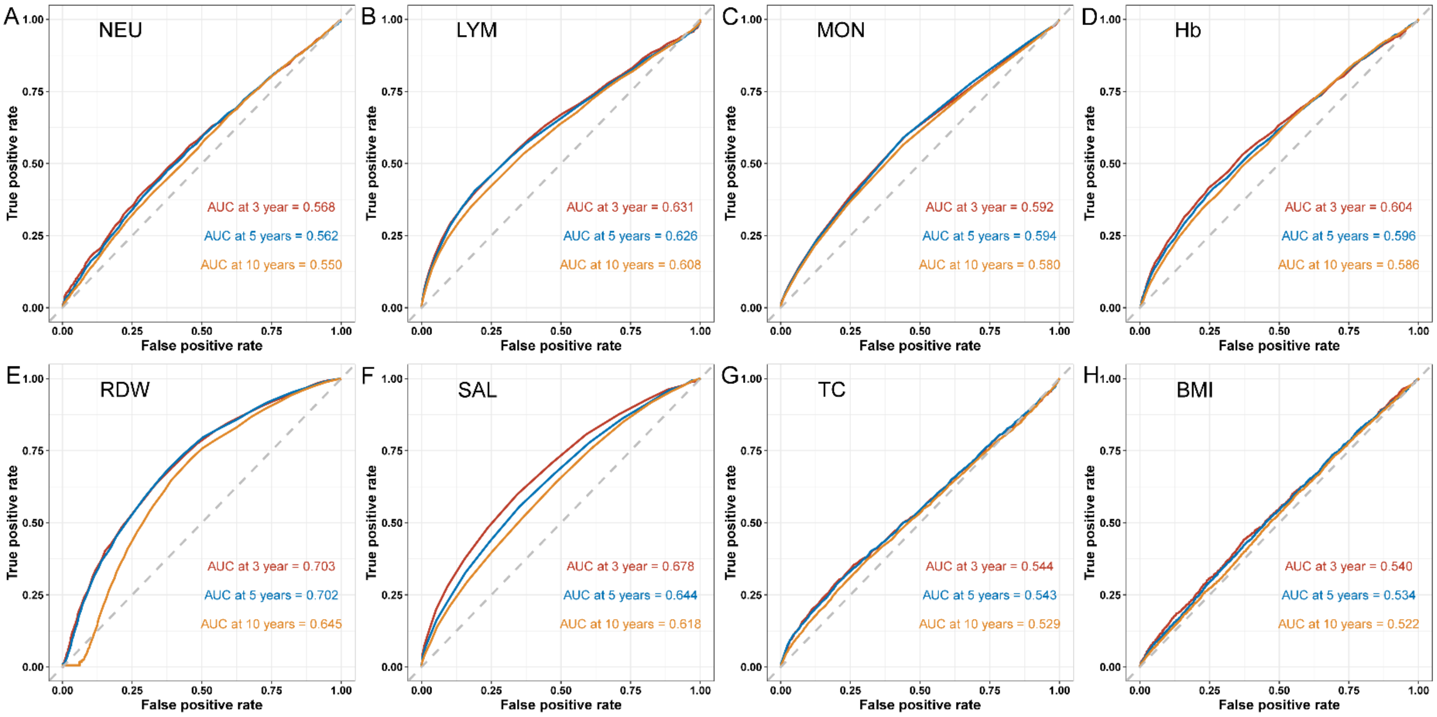

Figure S4. Predictive value of time-dependent ROC assessment of inflammation and nutritional indicators (A: NEU; B: LYM; C: MON; D: Hb; E: RDW; F: SAL; G: TC; and H: BMI) for 3-, 5-, and 10-year all-cause mortality. NEU, neutrophil; LYM, lymphocyte; MON, monocyte; Hb, hemoglobin; RDW, red cell distribution width; SAL, serum albumin; TC, total cholesterol; BMI, body mass index.

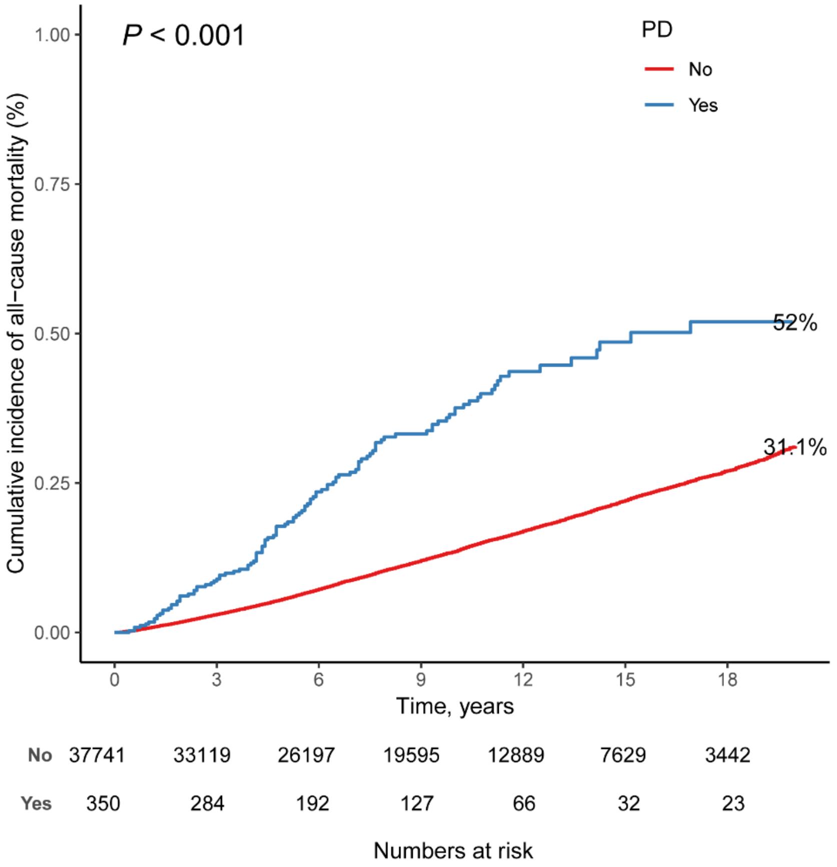

Figure S5. Kaplan-Meier survival curves for Parkinson's disease and mortality in adults.
